# Type D personality and social inhibition: drivers of cardiovascular risk and reduced physical activity in psoriatic arthritis

**DOI:** 10.1007/s10067-025-07312-3

**Published:** 2025-01-30

**Authors:** Ayşegül Yetişir, Aylin Sariyildiz, Ilke Coskun Benlidayi, Süleyman Özbek

**Affiliations:** 1 Division of Rheumatology, Department of Physical Medicine and Rehabilitation, Faculty of Medicine, Cukurova University, Adana, Turkey; 2 Department of Physical Medicine and Rehabilitation, Faculty of Medicine, Cukurova University, Adana, Turkey; 3 Division of Rheumatology, Department of Internal Medicine, Faculty of Medicine, Cukurova University, Adana, Turkey

**Keywords:** Framingham risk score, Metabolic syndrome, Psoriatic arthritis, Type D personality

## Abstract

**Background:**

To evaluate the presence of type D personality and its association with metabolic syndrome (MetS), cardiovascular disease risk, and level of exercise in patients with psoriatic arthritis (PsA).

**Material and method:**

This cross-sectional study included patients with PsA (*n* = 84) and healthy controls (*n* = 74). Sociodemographic data, laboratory parameters, and disease-related parameters were recorded. We evaluated the presence of type D personality with the total 14 items of the type D Personality Scale, the Framingham Risk Score (FRS) indicating 10-year cardiovascular disease risk as low, moderate, and high, the presence of MetS, and physical activity levels by the International Physical Activity Questionnaire-Short Form (IPAQ-SF).

**Results:**

The median age of patients with PsA was 53.5 (17) years, 73.8% were female, and the disease duration was 7 (12) years. Compared to controls, patients with PsA exhibited higher FRS and increased frequency of MetS, type D personality, and its domains (social inhibition and negative affectivity) (*p* < 0.05 for all). Patients with type D personality had even a higher frequency of MetS and lower levels of moderate-intensity exercise than those without type D personality (*p* = 0.020 and *p* = 0.027). Patients with social inhibition had higher FRS, a higher frequency of MetS, and lower levels of moderate-intensity exercise (*p* < 0.05 for all) compared to those without social inhibition. MetS showed a significant positive correlation with social inhibition, waist circumference, and FRS (Spearman’s rho were 0.244, 0.444, and 0.555, respectively), and a negative correlation with IPAQ-SF total metabolic equivalent (Spearman’s rho was -0.214).

**Conclusion:**

Patients with type D personality and social inhibition had a higher frequency of MetS and low levels of moderate-intensity exercise, whereas patients with social inhibition had a higher FRS. It is essential to assess PsA patients’ cardiac risk factors, type D personality, and social inhibition, as well as provide exercise advice.

**Table:** 

**Key Points** •*Psoriatic arthritis (PsA) is a prevelant rheumatologic disease that is frequently accompanied by comorbidities such as metabolic syndrome (MetS) and cardiovascular disease (CVD)*. •*It is crucial to evaluate patients with PsA for CVD risk factors*. •*Patients with type D personality demonstrate lower engagement in moderate-intensity exercise and a higher frequency of MetS. Socially inhibited PsA patients showed elevated Framingham Risk Scores and MetS rates, as well as reduced levels of moderate-intensity exercise*. •*Beyond assessing cardiac risk factors, it is important to assess personality traits and offer tailored exercise recommendations for comprehensive PsA management*.

## Introduction

Psoriatic arthritis (PsA) is a systemic autoimmune disease that causes inflammation in the enthesis, as well as peripheral and axial joints. Extra-articular manifestations such as psoriasis of the skin and nails, uveitis, and inflammatory bowel disease are common in PsA. It affects both men and women equally, predominantly within the 40–50 age range. PsA is accompained with comorbidities such as osteoporosis, metabolic syndrome (MetS), and cardiovascular disease (CVD). Although the pathophysiology is unclear, patients with PsA face a high risk of developing MetS due to overexpression of pro-inflammatory cytokines and the continuation of the inflammatory process [1, 2].

MetS is a condition characterized by disruptions in metabolic processes, such as insulin resistance, atherogenic dyslipidemia, abdominal obesity, and hypertension [3]. Patients with PsA have a high risk of CVD, as well as a high prevalence of MetS [2, 4]. In individuals with PsA, chronic inflammation is substantially related to increased rates of MetS and CVD [4, 5].

Personality traits in chronic diseases have garnered significant attention over the past decade, and type D personality has been associated with a number of clinical and social effects in various conditions such as CVD, fibromyalgia, and systemic lupus erythematosus [6–8]. There are numerous studies demonstrating the association between type D personality and CVD. Type D personality has been observed to have unfavorable outcomes on CVD prognosis [9, 10]. Social inhibition and negative affectivity are two characteristics that are associated with the type D personality. It describes a personality type that is vulnerable to negative emotions and tends to suppress self-expression in social interactions [11]. Some research in rheumatic diseases showed that type D personality can affect the clinical outcomes, such as poor quality of life and health parameters [12, 13]. On the other hand, the frequency of type D personality and the potential impact of personality traits on the development of MetS and/or CVD in PsA have not yet been identified. As far as we are concerned, this is the first study assessing type D personality and its association with conventional cardiovascular risk factors (e.g., MetS, 10-year CVD risk score, and exercise level) in patients with PsA. Given the above-mentioned lack in the literature, the aim of this study was to assess 1) the frequency of type D personality in patients with PsA, and 2) to identify the potential relation of this personality trait and its domains (social inhibition and negative affectivity) with MetS and CVD risk.

## Material and methods

### Study design

Between November 2023 and April 2024, this cross-sectional, single-center study was conducted at Cukurova University. The study included individuals diagnosed with PsA according to the classification criteria for PsA (CASPAR) [14], alongside a control group of otherwise healthy individuals who visited the outpatient clinic due to non-specific pain. Patients were excluded if they (i) were < 18 years and ≥ 65 years, (ii) had any other autoimmune rheumatic disease, (iii) had malignancy, (iv) suffered from severe psychiatric disorders, (v) had an acute or chronic infection, (vi) experienced major organ failure (e.g., cardiac, renal), and (vi) had alcohol and drug addiction.

### Sample size estimation

We calculated the sample size with the type D scale 14 (DS14) as the study variable [7]. The sample size was calculated as a minimum of 84 people for patients with PsA and 74 people for healthy controls, with a dispersion ratio of 0.86, statistical power of 0.95, and an alpha level of 0.05.

### Ethical issues

Local Ethics Committee of the Cukurova University approved the study protocol (Approval date: 03-November-2023, no: 138/25). Before commencing the study, each participant gave written informed consent. This study followed the principles of the Declaration of Helsinki.

### Study parameters

Sociodemographic details of the patients and the control group (e.g., age, years of education, etc.), body mass index (BMI), waist circumference (cm), and blood pressure (mmHg) were recorded. Laboratory parameters included C-reactive protein (CRP) (mg/dl), fasting blood glucose level (mg/dl), triglycerides (mg/dl), high-density lipoprotein cholesterol (HDLc) (mg/dl), low-density lipoprotein cholesterol (LDLc) (mg/dl), and total cholesterol (mg/dl) levels. Disease-related parameters of the patient group including duration of PsA, presence of extra-articular manifestations, count of tender and swollen joints, and medications (glucocorticoid, conventional synthetic Disease Modifying Anti-Rheumatic Drugs (csDMARD), biological DMARD (bDMARD), Janus kinase (JAK) inhibitors) were recorded. The disease activity index for PsA (DAPSA) [15] and the Charlson Comorbidity Index (CCI) were also documented for the patients.

Moreover, the study groups were assessed by the Framingham Risk Score (FRS) for the estimation of 10-year CVD risk [16] and the International Physical Activity Questionnaire-Short Form (IPAQ-SF) for determining physical activity levels. The presence of MetS was evaluated based on established criteria [17]. Type D personality was assessed by the DS14 self-report scale [18].

FRS include gender, age, total cholesterol level, HDLc level, systolic blood pressure, smoking, and the presence of diabetes mellitus (Step 1). Step 1 calculates the total points, and Step 2 determines the 10-year CVD risk, which is utilized to classify the patient's risk as low (≤ 10%), moderate (10–19%), or high (≥ 20%) [16].

The IPAQ-SF is a 7-item tool that gathers data on the frequency and duration of several types of physical activities, as well as time spent sitting. According to the IPAQ-SF scoring protocol, the total score is metabolic equivalent (MET)-minutes/week = 3.3 METs × walking minutes × walking days in the week plus 4 METs × moderate physical activity minutes × moderate physical activity days in the week plus 8 METs × vigorous physical activity minutes × vigorous days in the week.

MetS is usually diagnosed when the following ≥ 3 of 5 criteria are present: (i) waist circumference ≥ 102 cm in male and ≥ 88 cm in female, (ii) blood pressure ≥ 130/85 mmHg or use drug treatment for hypertension, (iii) triglyceride levels of ≥ 150 mg/dl or treatment for high triglyceride level, (iv) high fasting blood glucose level ≥ 100 mg/dl or medication for hyperglycemia, and (v) HDLc levels < 40 mg/dL in males, < 50 mg/dL in females [17].

The negative affectivity and social inhibition parts of the DS14 measure were designed to identify people with type D personality. The scale consists of a total of 14 items; each item is scored between 0 and 4. Type D personality is identified by a score of ≥ 10 points for the negative affectivity and social inhibition components [18–20].

## Statistical analyses

Statistical analyses were conducted using the Statistical Package for Social Sciences (version 26.0; SPSS, Chicago, IL). Descriptive statistics are presented as the mean ± SD, median (interquartile range; IQR), and number of patients (%) for normally distributed continuous, non-normally distributed continuous, and categorical variables, respectively. The sociodemographic data and study variables of the patients with PsA and controls were compared. Additionally, the clinical data of patients with PsA stratified for the presence of type D personality, social inhibition, and negative affectivity were compared by the Student’s *t*-test, the Mann–Whitney *U* test, Fisher’s exact, and Pearson’s chi-squared tests. The correlation of FRS, MetS, social inhibition, and negative affectivity with clinical variables was tested by Spearman’s correlation analysis. An alpha value < 0.05 was regarded as statistically significant.

## Results

The disease-related data of patients with PsA are shown in Table 1. The median disease duration was 7 (12) years. The median CCI score, NRS-pain, and DAPSA score were 1 (2), 3 (4), and 16 (20.4), respectively. According to DAPSA categorization, 10.7% of the patients were in remission, 33.3% had low, 33.3% had high, and 22.6% had very high disease activity. Of the patients, 70.2% were on csDMARDs and 40.5% were receiving bDMARD therapy (anti-TNF, IL-17/23 inhibitors) (Table 1).

**Table 1:** Disease-related variables of patients with PsA

	Patients with PsA (*n* = 84)
**Disease duration (years)**	7 (12)
**CCI**	1 (2)
**Count of tender joints**	1 (3)
**Count of swollen joints**	0 (0)
**NRS pain**	3 (4)
**CRP (mg/dl)**	5.3 (6.9)
**DAPSA**	16 (20.4)
**Categorization of DAPSA**	
Remission Low High Very high	9 (10.7%) 28 (33.3%) 28 (33.3%) 19 (22.6%)
**Extra-articular manifestations**	
Uveitis	2 (2.4%)
IBD	4 (4.8%)
Psoriasis	56 (66.7%)
Dactylitis	17 (20.2%)
Nail disease	21 (25%)
**Treatment**	
csDMARD Glucocorticoid Anti-TNF IL17 inhibitors IL23 inhibitors JAK inhibitors	59 (70.2%) 11 (13.1%) 13 (15.5%) 17 (20.2%) 4 (4.8%) 1 (1.2%)

Values are presented in *n* (%) or median (IQR)

*IQR* interquartile range, *PsA* psoriatic arthritis, *CCI* Charlson Comorbidity Index, *NRS* numeric rating scale, *CRP* C-reactive protein, *DAPSA* disease activity index for PsA, *IBD* inflammatory bowel disease, *cs* conventional synthetic, *DMARD* disease-modifying anti-rheumatic drugs, *anti-TNF* anti-tumor necrosis factor, *IL* interleukin, *JAK* Janus kinase

The comparison of study parameters between patients with PsA and controls revealed that age, gender, current smoking status, alcohol consumption, and laboratory parameters including HDLc, LDLc, and total cholesterol levels did not differ between groups (*p* > 0.05 for all) (Table 2). On the other hand, patients with PsA had higher BMI, waist circumference, triglycerides, fasting blood glucose, systolic and dyastolic blood pressure, and FRS than controls (*p* < 0.05 for all). Additionally, the frequency of MetS and type D personality was higher in patients with PsA than that in controls (*p* values were < 0.001 and 0.007, respectively) (Table 2).

**Table 2:** Comparative analysis of sociodemographic, laboratory, and other study parameters between patients with PsA and controls

	Patients with PsA (*n* = 84)	Control (*n* = 74)	*p*
**Age (years)**	53.5 (17)	51 (16)	0.141
**Gender**			
Female Male	62 (73.8%) 22 (26.2%)	54 (73%) 20 (27%)	0.905
**Marital status**			
Married Single	57 (67.9%) 27 (32.1%)	60 (81.1%) 8 (10.9%)	< 0.001
**Education (years)**	8 (6)	13 (4)	< 0.001
**BMI (kg/m**^**2**^**)**	30 ± 5.7	26.4 ± 4.5	< 0.001
**Current smoking ( +)**	34 (40.5%)	23 (31.1%)	0.220
**Package years**	0 (12)	0 (5)	0.068
**Alcohol consumption ( +)**	11 (13.1%)	11 (14.9%)	0.749
**Waist circumference (cm)**	100.7 ± 14.1	92 ± 12.8	< 0.001
**Laboratory parameters (mg/dl)**			
Triglycerides	139 (87)	105 (65)	0.004
HDLc	53.5 ± 14	57.6 ± 13.6	0.067
LDLc	122.7 ± 35.2	124.4 ± 34.7	0.760
Total cholesterol	206.7 ± 44.5	206.1 ± 47.1	0.929
Fasting blood glucose	97.5 (23)	90 (16)	0.013
**FRS**	14.6 (17.7)	5.3 (6.2)	< 0.001
**FRS risk level**			
Low Moderate High	36 (42.9%) 21 (25%) 27 (32.1%)	58 (78.4%) 12 (16.2%) 4 (5.4%)	< 0.001
**MetS**	45 (53.6%)	17 (23%)	< 0.001
**Type D personality**	28 (33.3%)	11 (14.9%)	0.007
**Social inhibition**	8 (11)	5 (8)	0.001
**Negative affectivity**	14.4 ± 8.3	6.9 ± 6.4	< 0.001
**Blood pressure (mmHg)**			
Systolic	133.6 ± 21.9	119.2 ± 11.3	< 0.010
Diastolic	80.5 (26)	75 (11)	< 0.001
**IPAQ-SF (MET)**			
Walking	216.2 (563.5)	371.3 (843.9)	0.117
Moderate	0 (0)	0 (30)	0.186
Vigorous	0 (0)	0 (0)	0.008
Total	302.2 (795.7)	636.9 (1250.8)	0.021
**Categorization of IPAQ-SF**			
Inactive Minimally active HEPA active	61 (72.6%) 18 (21.4%) 5 (6%)	39 (52.7%) 28 (37.8%) 7 (9.5%)	0.034

Values are presented in mean ± standard deviation, *n* (%), or median (IQR)

*IQR* interquartile range, *PsA* psoriatic arthritis, *BMI* body mass index, *HDLc* high-density lipoprotein cholesterol, *LDLc* low-density lipoprotein cholesterol, *FRS* Framingham Risk Score, *MetS* metabolic syndrome, *IPAQ-SF* International Physical Activity Questionnaire-Short Form, *MET* metabolic equivalent, *HEPA* health-enhancing physical activity

Comparative analysis of the study parameters between patients with and without type D personality are presented in Tables 3 and 4. Accordingly, the groups were statistically similar in terms of age, gender, BMI, marital status, smoking, alcohol consumption, disease characteristics, current medication, laboratory parameters, blood pressure, total IPAQ-SF score, median FRS score, and FRS risk level categorization (*p* > 0.05 for all) (Tables 3 and 4). Inversely, patients with type D personality had larger waist circumference and a higher frequency of MetS (*p* = 0.044 and *p* = 0.020, respectively). Based on the IPAQ-SF, the moderate-intensity physical exercise level was lower in patients with type D personality than that in patients with non-type D personality (*p* = 0.027) (Table 4).

**Table 3:** Comparative analysis of sociodemographic and disease-related parameters between patients with and without type D personality

	Type D personality (*n* = 28)	Non-type D personality (*n* = 56)	*p*
**Age (years)**	50 (16)	54 (16)	0.281
**Female gender**	21 (75%)	41 (73.2%)	0.861
**Education (years)**	5 (3)	11 (7)	0.001
**BMI (kg/m**^**2**^**)**	31.6 ± 4.7	29.2 ± 6	0.070
**Marital status**			
Married Single	19 (67.9%) 9 (32.1%)	38 (67.9%) 18 (32.1%)	0.491
**Current smoking ( +)**	12 (42.9%)	22 (39.3%)	0.753
**Package year**	0 (10)	0 (19)	0.991
**Alcohol consumption ( +)**	1 (3.6%)	10 (17.9%)	0.090
**Disease duration (years)**	10 (10.5)	5 (10.3)	0.059
**Count of tender joints**	1 (3)	1 (4)	0.575
**Count of swollen joints**	0 (0)	0 (0)	0.786
**CRP (mg/dl)**	4.7 (9.9)	5.8 (5.9)	0.673
**NRS pain**	2 (6)	4 (4)	0.288
**DAPSA**	12.4 (21.9)	17.5 (19.7)	0.396
**Categorization of DAPSA**			
Remission Low High Very high	4 (14.3%) 11 (39.3%) 6 (21.4%) 7 (25%)	5 (8.9%) 17 (30.4%) 22 (39.3%) 12 (21.4%)	0.417
**Extra-articular manifestations**			
Uveitis	0 (0%)	2 (3.6%)	0.550
IBD	0 (0%)	4 (7.1%)	0.296
Psoriasis	17 (60.7%)	39 (69.6%)	0.413
Dactylitis	5 (17.9%)	12 (21.4%)	0.701
Nail disease	6 (21.4%)	15 (26.8%)	0.593
**Treatment**			
csDMARD Glucocorticoid Anti-TNF IL17 inhibitors IL23 inhibitors JAK inhibitors	21 (75%) 5 (17.9%) 7 (25%) 5 (17.9%) 0 (0%) 0 (0%)	38 (67.9%) 6 (10.7%) 6 (10.7%) 12 (21.4%) 4 (7.1%) 1 (1.8%)	0.500 0.360 0.880 0.701 0.296 0.999

Values are presented in median (IQR), mean ± standart deviation, or *n* (%)

*IQR* interquartile range, *BMI* body mass index, *CRP* C-reactive protein, *NRS* numeric rating scale, *DAPSA* disease activity index for PsA, *IBD* inflammatory bowel disease, *cs* conventional synthetic, *DMARD* disease-modifying anti-rheumatic drugs, *anti-TNF* anti-tumor necrosis factor, *IL* interleukin, *JAK* Janus kinase

**Table 4:** Comparative analysis of study parameters between patients with type D personality and non-type D personality

	Type D personality (*n* = 28)	Non-type D personality (*n* = 56)	*p*
**CCI**	1 (3)	1 (2)	0.490
**Waist circumference (cm)**	105 ± 12.6	98.5 ± 14.4	0.044
**Laboratory parameters (mg/dl)**			
Triglycerides	155 (103)	129.5 (89)	0.091
HDLc	49.4 ± 9.7	55.6 ± 15.4	0.052
LDLc	123.2 ± 24.9	122.4 ± 39.5	0.924
Total cholesterol	204.6 ± 40.9	207.8 ± 46.6	0.759
Fasting blood glucose	96.5 (25)	98 (23)	0.883
**FRS**	17.1 (23.7)	10 (17.5)	0.208
**FRS risk level**			
Low Moderate High	9 (32.1%) 7 (25%) 12 (42.9%)	27 (48.2%) 14 (25%) 15 (26.8%)	0.269
**MetS**	20 (71.4%)	25 (44.6%)	0.020
**Blood pressure (mmHg)**			
Systolic	137 ± 23	131.86 ± 21.4	0.310
Diastolic	85 (24)	80 (19)	0.345
**IPAQ-SF (MET)**			
Walking Moderate	151.8 (712) 0 (0)	282.2 (563.5) 0 (0)	0.920 0.027
Vigorous	0 (0)	0 (0)	0.547
Total	158.4 (787.8)	347.4 (850)	0.516

Values are presented in median (IQR), mean ± standart deviation, or *n* (%)

Abbreviations: *IQR* interquartile range, *CCI* Charlson Comorbidity Index, *HDLc* high-density lipoprotein cholesterol, *LDLc* low-density lipoprotein cholesterol, *FRS* Framingham Risk Score, *MetS* metabolic syndrome, *IPAQ-SF* International Physical Activity Questionnaire-Short Form, *MET* metabolic equivalent

Comparative data according to the presence of social inhibition and negative affectivity in patients with PsA are displayed in Table 5. Accordingly, patients with social inhibition had higher FRS and a higher frequency of MetS, compared to those without social inhibition. Socially inhibited patients also exhibited higher triglyceride levels and lower levels of HDLc. They also showed lower MET value of moderate-intensity physical activity (*p* < 0.05 for all).

**Table 5:** Comparison based on the presence of social inhibition and negative affectivity in patients with PsA

	Social inhibition ( −) (*n* = 49)	Social inhibition ( +) (*n* = 35)	*p*	Negative affectivity ( −) (*n* = 27)	Negative affectivity ( +) (*n* = 57)	*p*
**Age** (years)	54 (17)	52 (16)	0.982	54 (14)	53 (19)	0.777
**Education** (years)	11 (9)	5 (6)	0.003	11 (10)	8 (6)	0.012
**BMI** (kg/m^2^)	29.7 ± 6.1	30.5 ± 5.1	0.504	29.9 ± 6.8	30.1 ± 5.2	0.911
**Package year**	0 (18)	0 (10)	0.959	0 (6)	0 (16)	0.174
**Disease duration** (years)	5 (8)	10 (11)	0.073	5 (9)	8 (11)	0.187
**CCI**	1 (2)	1 (3)	0.285	1 (2)	1 (3)	0.403
**Tender joint count**	1 (4)	1 (3)	0.872	1 (3)	1 (4)	0.352
**Swollen joint count**	0 (0)	0 (0)	0.773	0 (0)	0 (0)	0.831
**NRS pain**	4 (4)	2 (5)	0.577	4 (5)	3 (5)	0.988
**CRP** (mg/dl)	6 (6.5)	5 (8)	0.406	5.4 (6.6)	5.1 (7.7)	0.355
**DAPSA**	17 (19)	15.3 (22.2)	0.634	18.2 (21.8)	14.5 (20.7)	0.882
**Waist circumference** (cm)	99.2 ± 14.8	102.7 ± 13.1	0.270	99.5 ± 15.6	101.2 ± 13.5	0.599
**Laboratory parameters (mg/dl)**						
Triglycerides HDLc LDLc Total cholesterol Fasting blood glucose	126 (89) 56.8 ± 15.9 119.4 ± 33.9 204.9 ± 41.8 98 (22)	161 (107) 49.1 ± 9.5 127.2 ± 36.9 209.3 ± 48.6 96 (24)	0.027 0.007 0.319 0.659 0.845	136 (102) 54.7 ± 13.6 126.9 ± 46.6 212.7 ± 50.8 98 (23)	140 (87) 53 ± 14.3 120.7 ± 28.5 203.9 ± 41.4 97 (22)	0.811 0.595 0.525 0.401 0.981
**Blood pressure (mmHg)**						
Systolic Diastolic	131.5 ± 22.1 80 (21)	136.5 ± 21.7 84 (25)	0.303 0.374	135 ± 23.2 82 (16)	132.9 ± 21.5 80 (28)	0.694 0.950
**MetS ( +)**	21 (42.9%)	24 (68.6%)	0.020	13 (48.1%)	32 (56.1%)	0.493
**FRS**	8.6 (16.7)	18.4 (21.2)	0.036	13.2 (16.8)	15.9 (19.6)	0.784
**IPAQ-SF (MET)**						
Sedentary (hours) Walking Moderate Vigorous Total	5 (5) 330 (635.4) 0 (58.7) 0 (0) 412.5 (735.5)	6 (5) 138.6 (462) 0 (0) 0 (0) 632.3 (1213)	0.340 0.296 0.031 0.956 0.230	5 (5) 247.5 (635.3) 0 (0) 0 (0) 304 (1027.9)	5 (5) 198 (549.5) 0 (0) 0 (0) 300.3 (696)	0.519 0.931 0.295 0.021 0.689

Values are presented in median (IQR), mean ± standard deviation, or *n* (%)

*PsA* psoriatic arthritis, *BMI* body mass index, *CCI* Charlson Comorbidity Index, *NRS* numeric rating scale*, CRP* C-reactive protein*, DAPSA* disease activity index for PsA, *HDLc *high-density lipoprotein cholestrol, *LDLc* low-density lipoprotein cholesterol, *MetS* metabolic syndrome, *FRS* framingham risk score, *IPAQ-SF* international physical activity questionnaire-short form, *MET* metabolic equivalent

Table 6 presents the outcomes of the correlation analysis between the study variables. A significant positive correlation (Spearman’s rho ranged from 0.292 to 0.720) was found between FRS and the package year, BMI, triglycerides, waist circumference, age, and CCI. The presence of MetS showed a positive correlation with social inhibition, BMI, age, waist circumference, triglycerides, FRS, and CCI (Spearman’s rho ranged from 0.244 to 0.567). FRS and MetS were negatively correlated with IPAQ-SF total MET (Spearman’s rho = − 0.220 and − 0.214, respectively).

**Table 6:** Correlation of study variables in patients with PsA

	FRS	MetS	Social inhibition	Negative affectivity
**Age** (years)	0.596***	0.403***	− 0.040	− 0.019
**Education** (years)	− 0.377***	− 0.496***	− 0.374***	− 0.266*
**BMI** (kg/m^2^)	0.298**	0.363***	0.101	0.033
**Package year**	0.292**	− 0.171	− 0.025	0.160
**Disease duration** (years)	0.146	0.208	0.200	0.108
**CCI**	0.720***	0.567***	0.083	0.120
**DAPSA**	0.100	0.068	− 0.078	− 0.004
**Waist circumference** (cm)	0.427***	0.444***	0.180	0.043
**Triglycerides** (mg/dl)	0.352**	0.553***	0.236*	0.024
**FRS**	-	0.555***	0.203	0.004
**Social inhibition**	0.203	0.244*	-	0.349**
**Negative affectivity**	0.004	0.045	0.349**	-
**IPAQ-SF** **total** (MET)	− 0.220*	− 0.214*	− 0.048	− 0.069

*ρ* values represent Spearman’s rho

^*^p < 0.05, **p < 0.01, ***p < 0.001

*PsA* psoriatic arthritis, *FRS* Framingham Risk Score, *MetS* metabolic syndrome, *BMI* body mass index, *CCI* Charlson Comorbidity Index, *DAPSA* disease activity index for PsA, *IPAQ-SF* International Physical Activity Questionnaire-Short Form, *MET* metabolic equivalent

## Discussion

The current study showed that patients with PsA had a higher 10-year CVD risk and MetS frequency. Type D personality was also more common in the patients with PsA. Patients with type D personality had a greater frequency of MetS than those with non-type D personality. They also reported lower levels of moderate-intensity exercise. Moreover, socially inhibited patients exhibited a higher 10-year CVD risk, MetS frequency, and triglyceride levels; lower HDLc and moderate-intensity physical activity levels. Previous studies have reported increased FRS and an increased incidence of MetS in patients with PsA. Although literature points an elevated cardiac risk in individuals with type D personality, cardiovascular risk in PsA patients with type D personality has not been reported so far. The current study provides a novel insight to the point by investigating the relation of type D personality and its determinants with MetS and FRS in PsA.

The underlying pathophysiology of PsA, MetS, and atherosclerosis may have overlapping inflammatory pathways and genetic predispositions. Patients with PsA have a high frequency of MetS, which ranges from 29.1% to 56% [21–24]. Cardiovascular risk is also high in PsA [25–27]. MetS is a well-known factor that increases the risk of atherosclerosis and CVDs in patients with PsA [4]. In line with the literature, we detected higher MetS components (except for HDLc) and increased cardiovascular risk in the PsA group than those in the control group. Over 50% of the patients revealed MetS and FRS was 14.6 in the patient group.

The association of type D personality with cardiac diseases has been reported several times [9, 10, 28]. Although the underlying pathophysiology of the relationship between type D personality and CVDs is not well understood, it is assumed that sympathetic-adrenal-medullary system and hypothalamic-pituitary-adrenocortical dysfunction might play a role [29]. Kim et al. reported that the rate of MetS was higher in individuals with type D personality (13% vs. 6.4%). Higher negative affectivity and social inhibition subsets were correlated with MetS and CVD severity [30]. In the current study, we found that the frequency of MetS was higher in PsA patients with type D personality. Those patients also exhibited lesser amounts of moderate-intensity physical exercise compared to non-type D counterparts. This finding is consistent with the current knowledge that individuals with type D personality are less likely to spend time in exercise and tend to perform more sedentary activities [31, 32].

In order to prevent CVDs, the American Heart Association recommends moderate or vigorous activity for individuals. Low- to moderate-intensity physical activity has beneficial effects on endothelial function, insulin resistance, cardiac remodeling, and lipid metabolism [33]. For adults, Health Canada recommends daily moderate activity (3–6 MET) [34]. Moderate-intensity activity is also recommended for adults with MetS [35]. Although the American Heart Association recommends vigorous activity to prevent CVDs, it has some disadvantages, such as high mortality and sudden death, and requires expert supervision and guidance [33]. Exercising vigorously may also increase the risk of harm and decreases compliance [34]. Harmful effects (e.g., joint destruction) of vigorous physical activity may be more profound in patients with acute arthritis. Patients with autoimmune inflammatory rheumatic diseases are recommended to exercise in a careful fashion in order to ensure joint protection [36]. Overall, an individual-based exercise program can help lower the risk of CVDs in autoimmune inflammatory rheumatic conditions by reducing prolonged inflammatory status, improving vascular function and lipid profile, restoring the autonomic system, and increasing muscular function [37]. Yet, there are numerous patient-related and/or environmental variables that might hinder patients from engaging in regular physical activity. In the current study, we determined the role of type D personality and social inhibition as potential determinants of lower levels of moderate-intensity physical activity, which could contribute to an increased risk of CVD in PsA. Such parameters, therefore, should be considered meticulously in the management of PsA.

Prolonged or recurrent stressors, such as type A and D personalities, work stress, and financial problems can cause chronic stress, which can accelerate atherosclerosis and increase the risk of coronary artery disease [38]. Social inhibition exposes type D individuals to stress in social situations. Social inhibition and negative affectivity have been reported to raise blood cortisol levels. Coronary artery calcification, elevated blood pressure, and CVD risk are also associated with increased cortisol reactivity [30, 39]. The results of the current study revealed a close relationship between social inhibition and cardiac risk factors (e.g., MetS). Socially inhibited individuals also appeared to less likely engage in moderate-intensity exercises, which could be regarded as a potential factor increasing the cardiovascular risk in patients with PsA.

This cross-sectional study has some limitations and strengths. One of its limitations is that the questionnaires utilized may be biased because of being subjective. Another limitation is that the IPAQ-SF questionnaire only measures exercise levels within the last week. The questionnaire, therefore, might not have reflected overall exercise habits accurately. Furthermore, it is difficult to exclude all confounding factors, probably interfering with the analysis and results. A pivotal strength of this study is its exhaustive analysis of the potential influence of type D personality and its subdomains on MetS, cardiac risk score, and exercise levels in patients with PsA.

In conclusion, one-third of the patients with PsA exhibited type D personality. MetS was more frequent in patients with this personality trait. Social inhibited patients had higher percentage of MetS and cardiovascular risk scores. Patients with social inhibition and/or type D personality failed to achieve the recommended moderate-intensity exercise levels to lower their cardiovascular risk. It is important to evaluate patients with PsA in terms of cardiac risk factors, type D personality, social inhibition, and habits of exercise. Patients should be provided with adequate social support and personalized exercise recommendations to address their needs effectively. To better understand these associations, further research involving larger patient cohorts and long follow-up periods is necessary.

## Data Availability

The datasets gathered during the preparation of this manuscript are available from the corresponding author upon reasonable request.
